# Editorial: Natural Antibodies in Health and Disease

**DOI:** 10.3389/fimmu.2017.01795

**Published:** 2017-12-13

**Authors:** Ana Maria Hernandez, Nichol E. Holodick

**Affiliations:** ^1^Natural Antibodies Group, Tumor Immunology Division, Center of Molecular Immunology, Havana, Cuba; ^2^Department of Biomedical Sciences, Center for Immunobiology, Western Michigan University Homer Stryker MD School of Medicine, Kalamazoo, MI, United States

**Keywords:** natural antibodies, B cells, B-1 cells, antibodies, natural antibodies repertoire

**Editorial on the Research Topic**

**Natural Antibodies in Health and Disease**

Natural antibodies (NAbs) are most commonly defined as immunoglobulins present in the absence of exogenous antigen stimulation. In fact, numerous groups have demonstrated the presence of NAbs in both specific pathogen-free and germ-free mice (1–3). These NAbs provide immediate protection against infection while the adaptive arm of the immune system mounts a specific and long-lasting response. Beyond immediate protection from infection, NAbs have been shown to play various functional roles in the immune system, which include clearance of apoptotic debris (Gronwall et al.), suppression of allergic responses (4, 5), regulation of B cell responses (6), selection of the B cell repertoire (7, 8), protection from cancer (9, 10), regulation of B cell development [Baumgarth; (7, 11)], and protection against atherosclerosis (12–15). These various functions of NAbs are afforded by their reactivity, which is broad, cross-reactive, and shown to recognize evolutionarily fixed epitopes present in foreign antigens [Gronwall et al.; (16–21)]. Furthermore, NAbs have unique characteristics that also contribute to their functional roles and set them apart from antigen-specific antibodies. Such characteristics include germline structure (lacking non-templated nucleotides and little to no somatic hypermutation) and a restricted repertoire (16, 22–24).

Determining and subsequently examining the B cells producing NAbs have been the subject of intense investigation since the early 1980s despite NAbs being studied since the late 1960s. NAb producing B-1a cells were first identified in mice and characterized by surface expression of CD5^+^, IgM^high^, IgD^low^, CD19^high^, B220^low^, CD23^−^, and CD43^+^ (25), which contrasts with the surface phenotype of follicular B-2 cells: CD5^−^, IgM^low^, IgD^high^, CD19^+^, B220^+^, CD23^+^, and CD43^−^. Studies have demonstrated that B-1a cells are found in the peritoneal cavity, pleural cavity, spleen, bone marrow, lymph nodes, and blood of mice (26). Furthermore, various subsets of B-1a cells have been identified and include those expressing PD-L2 (PD-L2^+/−^) (27, 28), CD25 (CD25^+/−^) (Tumang et al.), CD73 (CD73^hi/lo^) (29), and PC-1 (PC-1^hi/lo^). Throughout the many years of B-1a cell investigation, it has been shown that not all subsets of murine B-1a cells secrete NAbs. This has important implications when investigating the source of protective and/or pathogenic NAbs.

More recently, focus has been given in determining the subset of B cells in humans capable of producing NAbs. Early studies in humans focused on CD5^+^ peripheral B cells (30–33) and CD5-CD45RAlo B cells (34). More recently, attempts to refine the human NAb producing B cell subset have generated a new phenotypic definition: CD20^+^CD27^+^CD43^+^CD70^−^CD38^mod^ (35, 36). Interestingly, most of these cells express CD5. Further investigation is still required in the human system to determine whether other subsets of NAb producing B cells exist and the location of such subsets beyond peripheral blood.

Many aspects of NAbs and the cells generating them have yet to be studied in great detail: the reactivity and function of NAbs in health and different diseases, the behavior of the NAb repertoire with increasing age, the regulation of NAb production and auto-reactivity, the ways to specifically activate NAbs producing B-1 cells with desired specificities, and the characteristics of human NAbs, among others. This Frontiers research topic aimed to further investigate how NAbs are regulated, the cells that generate NAbs, and the roles NAbs play in maintaining health and/or leading to disease.

The 16 articles presented in this research topic explore a wide range of topics pertaining to NAbs (and the cells that produce them) in health and disease. These papers investigate the specificity of NAbs [Cruz-Leal et al.; Vale et al.; Zhang et al.], the function of NAbs [Pedersen et al.; Kohler et al.; Rothstein; Saha et al.], the cells producing NAb [Baumgarth; Popi et al.; Kaku et al.], and/or the role NAbs and/or NAb producing cells play in leading to disease [Wang et al.; Holodick et al.; Lobo; Wolfram et al.; Zhu et al.]. In addition, we include a perspective article aiming to start discussion and investigation into the definition of NAbs (Holodick et al.). With the plethora of established and new data on NAbs and NAb producing cells, it is clear our traditional definition of such antibodies might need to be refined or bolstered. Overall, this collection of articles adds to the NAb literature in a thoughtful and hopefully thought-provoking way. We thank all of the authors for contributing their work to this ebook, which will inspire many new lines of investigation into the structure, generation, and function of NAbs in health and disease.

## Author Contributions

Both authors contributed equally to the writing of this editorial.

## Conflict of Interest Statement

The authors declare that the research was conducted in the absence of any commercial or financial relationships that could be construed as a potential conflict of interest.
